# Neural network embedding of functional microconnectome

**DOI:** 10.1162/netn_a_00424

**Published:** 2025-03-05

**Authors:** Arata Shirakami, Takeshi Hase, Yuki Yamaguchi, Masanori Shimono

**Affiliations:** Graduate Schools of Medicine, Kyoto University, Kyoto, Japan; The Systems Biology Institute, Tokyo, Japan; Center for Education in Healthcare Innovation, Institute of Science Tokyo, Tokyo, Japan; SBX BioSciences, Inc., Vancouver, BC, Canada; Faculty of Pharmacy, Keio University, Tokyo, Japan; Center for Mathematical Modelling and Data Science, Osaka University, Osaka, Japan; Hakubi Center, Kyoto University, Kyoto, Japan; Graduate School of Information Science and Technology, Osaka University, Osaka, Japan

**Keywords:** Network embedding, Neural networks, Centrality, New metrics, Indirect-adjacent degree, Neighbor hub ratio, Microconnectome

## Abstract

Our brains operate as a complex network of interconnected neurons. To gain a deeper understanding of this network architecture, it is essential to extract simple rules from its intricate structure. This study aimed to compress and simplify the architecture, with a particular focus on interpreting patterns of functional connectivity in 2.5 hr of electrical activity from a vast number of neurons in acutely sliced mouse brains. Here, we combined two distinct methods together: automatic compression and network analysis. Firstly, for automatic compression, we trained an artificial neural network named NNE (neural network embedding). This allowed us to reduce the connectivity to features, be represented only by 13% of the original neuron count. Secondly, to decipher the topology, we concentrated on the variability among the compressed features and compared them with 15 distinct network metrics. Specifically, we introduced new metrics that had not previously existed, termed as indirect-adjacent degree and neighbor hub ratio. Our results conclusively demonstrated that these new metrics could better explain approximately 40%–45% of the features. This finding highlighted the critical role of NNE in facilitating the development of innovative metrics, because some of the features extracted by NNE were not captured by the currently existed network metrics.

## Author Summary

Neural network embedding can compress large connectivity and has led to new metrics like indirect-adjacency degree and neighbor hub ratio.

## INTRODUCTION

### Network Architecture of Neuronal Circuits

Our brain allows us to perform a myriad of functions based on activation patterns within complex structural networks. If we can extract the underlying organizational rules behind the design of these networks, we can better understand how various functions manifest within the system. From this vantage point, quantitative evaluations of brain network variables have been conducted across different spatial scales (Fornito et al., 2016; Sporns, 2016).

Studying the brain from a macroscopic perspective, structural connections (white matter fiber bundles) link elements corresponding to brain regions. The effective number of brain regions comprising the macroconnectome is in the range of 102–103 (e.g., Zalesky et al., 2010), provided that voxel-based analyses are not employed. Thus, characterizing community architectures and influential nodes is not presently challenging, especially when compared with gene networks, protein networks, biomedical networks, social networks, and others (Bales & Johnson, 2006; Radicchi et al., 2004).

However, recent trends in research are leaning toward microconnectomes, where cells are considered as network elements (Reimann et al., 2019; Schroeter et al., 2015; Shimono & Beggs, 2015). Currently, network variables in neuroscience are derived directly from the original networks without dimension compression, as the number of neurons is still around 103–104 in many cutting-edge studies. However, the number of simultaneously recordable neurons has been doubling every year for the past 7 years since 1970 (Hong & Lieber, 2019; Stevenson & Kording, 2011). As these neuronal network sizes grow, effective compression becomes increasingly crucial to extract interpretable architecture embedded in the very high dimensional data. Consequently, there is an imperative to devise new analytical schemes anticipating future technological advances. The subsequent subsections delve into the narratives leading to the evolution of analytical schemes for data typified as networks.

### How Do We Compress Information?

#### Network variables.

With the recent expansion of big data in complex networks, including social networks, gene regulatory networks, and real neural networks, various network metrics have been developed to investigate the statistical and topological characteristics of these networks (Barabási & Oltvai, 2004; Borgatti et al., 2018). In prior network neuroscience studies, these metrics have been employed to characterize the network architectures of individual neural systems (Bassett & Sporns, 2017; Rubinov & Sporns, 2010). Broadly speaking, metrics such as centrality metrics, clustering, and community structure characterize different scales of the topological architectures of network systems.

Firstly, centrality metrics identify important nodes in the network from various perspectives (e.g., degree, k-core, page rank, subgraph, etc.). They provide insight into the centrality of an individual node within the broader network pattern (Bavelas, 1948; Borgatti & Everett, 2006; Brin & Page, 1998; Estrada & Rodríguez-Velázquez, 2005; Freeman, 1978; Sabidussi, 1966; Seidman, 1983).

Secondly, clustering coefficients and network motifs pertain to small groups of nodes. They characterize the statistical frequency of specific connectivity patterns that occur more often than expected by chance (Milo et al., 2002; Watts & Strogatz, 1998).

Thirdly, community architectures are about global groups of nodes with strong interconnections. These groups are defined based on various criteria (Girvan & Newman, 2002; Guimerà & Amaral, 2005; Kawamoto, 2018; Lancichinetti & Fortunato, 2009). After segmenting networks into communities, the participation coefficient assesses the likelihood of individual nodes belonging to multiple communities.

These traditional approaches have the advantage of making it relatively easy to interpret network characteristics by quantifying individual metrics. These metrics also have solid mathematical foundations. They were developed based on the focused intentions of past researchers aiming to understand specific facets of target systems described as complex networks.

However, despite the merits of these evaluation schemes, it is crucial to note that there is no guarantee that existing metrics are optimal for characterizing newly produced datasets. Another key observation is that biological system networks, including the brain, are not solely determined by the optimality indicated by individual metrics (Chklovskii, 2004). For instance, while connection arrangements like preferential attachment and spatially proximate connectivity influences are factors, they do not singularly dictate network design. Instead, their interplay determines the network’s structure (Nicosia et al., 2013; Vértes et al., 2012). Understanding a network holistically, where multiple metric-induced characteristics balance out, demands more than computing each metric in isolation. Data-driven analytical methods for deconstructing these components prove effective (Betzel et al., 2016; Henriksen et al., 2016). Furthermore, when analyzing unfamiliar network architectures, it is vital to determine the need for additional, unprepared metrics.

Broadening our perspective, extracting features that naturally and optimally match the characteristics of individual datasets, without a researcher’s bias toward specific network metrics, is now a central interest in modern neural network analyses.

#### Neural network embedding (NNE) by artificial neural networks.

Recently, researchers have adopted network embedding approaches that leverage various data compression techniques to automatically extract features from large complex networks. Among these, “Deep Autoencoders” are a subset of deep neural networks that have historically been used. For example, among deep learning technologies, Perozzi et al. (2014) developed “DeepWalk,” which combined short-step random walks with SkipGram modeling. Following this, Grover and Leskovec (2016) introduced “node2vec,” which enhanced DeepWalk using efficient node sampling techniques. Their capabilities have been significantly enhanced thanks to the recent advances in neural network technology. A deep autoencoder comprises multiple “encoder” and “decoder” layers. The encoder layers compress the original data into a reduced feature space, while the subsequent decoder layers reconstruct the original data from these compressed features.

The weights of the links connecting nodes in two different layers are optimized to minimize discrepancies between the input and output layers. After this optimization process, the encoder layer with the fewest nodes (commonly referred to as the middle encoder layer) provides a compressed representation that captures essential features embedded within the original, nonlinear, complex data (Hinton & Salakhutdinov, 2006). As this method allows us to embed the original connectivity data into a compressed feature space using a neural network, we refer to this approach as “NNE” throughout this manuscript.

A limitation of this method is its reduced interpretability in mathematical terms, a trade-off for the benefits of automation. Therefore, to elucidate the significance of the features extracted via the NNE approach, it is imperative to compare these features not only with existing network metrics but also with newly developed network metrics.

### The Main Target of This Study

In this study, we employed one of the training models of artificial neural networks, considered as an NNE method (Cao et al., 2016; Wang et al., 2016), to compress the real data of neural connectivity networks into a smaller, recoverable format. To our knowledge, there are no prior instances of NNE being applied to actual neuronal interaction networks.

We demonstrated that our method can compress a network architecture, originally comprising 100 neurons in a given dataset, down to 13 distinct features. This means that the original connection patterns in these real neuronal interaction networks, which we also term as “effective networks,” can be reconstructed from a mere 13% of the compressed space.

Given that prior research has shown that effective connectivity network architectures of neuronal microcircuits contain highly central hubs (Gal et al., 2017; Kajiwara et al., 2021; Nigam et al., 2016; Schroeter et al., 2015; Shimono & Beggs, 2015), our study sought to determine whether this focal point—namely, that centrality might be a critical feature for characterizing network architecture—holds true when analyzed using an unbiased, automatic extraction technique. Hence, we juxtaposed the features, compressed in a data-driven manner by the NNE method, with widely utilized network metrics, predominantly various centrality metrics, along with several noncentrality metrics. Moreover, we devised new network metrics for comparison to address the limitations of conventional metrics, revealing that these new metrics aptly explained 40%–45% more features derived from NNE than the traditional ones.

A pivotal point to underscore is the complementary nature of NNE and network metrics. While NNE excels at automatic information compression, it does not inherently offer an interpretation for the compressed features. Thus, the significance of these compressed features becomes evident only when juxtaposed with network metrics. In our study, we compared the 13 features extracted by NNE against 15 network metrics, resulting in a consideration of 13 × 15 feature combinations.

**Figure 1.**
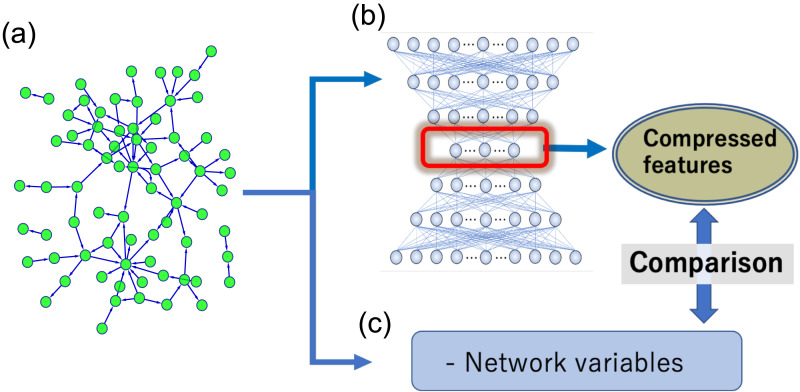
The main logical structure of this study: (A) shows an example of interaction networks among neurons. How we extract compressed features of such networks is the main question that this study asks. (B) The NNE scheme naturally compresses original big networks to a small space through optimizing weights of NNE to the raw connectivity network data. (C) We compared the compressed expression (features) with representative network metrics (e.g., degree, betweenness centrality, subgraph centrality, participation coefficient, clustering coefficient, and new network metrics given in this study). We defined and named the two new network metrics, indirect-adjacent degree and neighbor hub ratio, to improve the explainability of the NNE-based analysis.

Furthermore, we also assessed the recovery level of slightly randomized data processed through NNE, which had been trained on the original nonrandomized data, to establish the reliability of data recovery from the 13 features (Figure 1).

## MATERIALS AND METHODS

### Physiological Recording of Neuronal Activities

We utilized neuronal spike data recorded and extensively analyzed in our previous study. A brief explanation of the experimental procedure from this past study can be found in Kajiwara et al. (2021). The complete experimental processes are also openly accessible in a video journal (Ide et al., 2019). We worked with seven female C57BL/6 J mice (*n* = 7, aged 3–5 weeks), collecting a total of 15 data samples, with two to three samples obtained from each mouse. All animal procedures adhered to Kyoto University (KU) guidelines for animal experiments and received approval from the KU Animal Committee.

**Figure 2.**
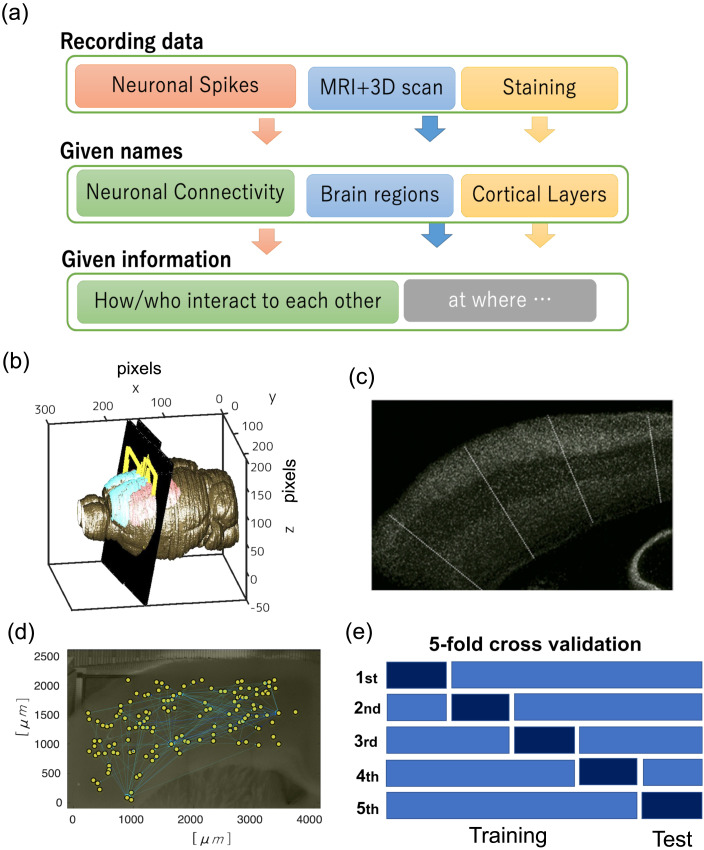
(A) Schematic flow of the experimental dataset given from Kajiwara et al. (2021). Refer to the Supporting Information about the experimental procedure in detail. The first row at the top lists the three types of data we used. They consist of electrical activity of neurons, MRI data (+3D data scans), and staining data. The second and third columns from the top show the given names (categories of physiological targets) and information provided by analyzing the given data, respectively. The second row lists the physiological or anatomical information that the three types of data provide respectively, and they are covered with the three different colors that correspond to the top row. The third column shows “how,” “who,” and “where” the type of information was provided by analyzing the given data. We were able to know how (as a central position in the network, or as making clustering patterns with other several nodes etc.) and who (excitatory or inhibitory neurons) interact with each other at where (a layer in a brain region). We can get information on anatomical regions (or spatial areas) by integrating MRI data (+3D data scan) and staining data. In other words, the “where” information can be obtained only by integrating the two items listed above, and thus is given a new color. (B) The black slices and yellow regions in the three dimensional brain volumes depict the two dimensional brain surfaces where we recorded electrical activity. (B) This image shows how we are able to know the recording brain region from MRI and slicing position recorded with a 3D scan. (C) The white dots are neurons detected with immunostaining, and we are able to find striped patterns in the cortical slice. The pattern is called cortical layers. As we also mentioned in the main document, please notice the meaning of “layer” is different from the number of layers used for defining our artificial neural networks utilized as the analyzing side. As shown as white lines on the cortical slice image, we divided neurons into small subsets holding just 100 neurons, and produced 15 datasets. Because the dividing lines are selected vertically against the layer’s boundaries, we are able to include all layers into all datasets. (D) An example of a connectivity network among neurons plotted on a photo image, in which nodes and links represent neurons and interactions between pairs of neurons, respectively (see Defining connectivity reflecting neuronal interactions section for details). (E) Then, we performed a fivefold cross validation with separating the 15 datasets to training and test data. Individual test data included three datasets. Among the 12 datasets in training data, we used three datasets as validation data that were also used for early stopping evaluation.

In this study, neuronal spikes were recorded from 300-μm-thick cortical slices using the cutting-edge multi-electrode array (MEA) system (Maxwell Biosystem, MaxOne). This was done while recirculating an artificial cerebrospinal fluid solution saturated with 95% O_2_/5% CO_2_ (Ide et al., 2019; Kajiwara et al., 2021). The slicing positions were meticulously controlled within the somatomotor areas. This precision was achieved by comparing 3D scan images of brain surfaces—captured immediately post brain extraction—with MRI images taken prior to the extraction (Figure 2A). Morphologically, the cerebral cortex—being the most recent evolutionary brain region—resembles a sheet that envelops all other brain regions. Its surface pattern in the depth direction can be likened to the overlapping of multiple sheets, similar to the concentric layers seen in tree rings.

Experimentally, we identified the cortical layers in offline imaging processes by referencing NeuN immunostaining images. In our artificial neural network, termed NNE, the depth of the artificial cells from input to output is also commonly referred to as a “layer.” It is essential to note, however, that these cortical layers represent an entirely different concept.

Moreover, spike sorting was executed (using the Spyking Circus software) to identify approximately 1,000 neurons from the electrical signals initially associated with individual electrodes (Ide et al., 2019; Kajiwara et al., 2021). The short intervals (15 μm) between the electrodes in the MEA system allowed for a highly accurate estimation of the spatial positions of the neurons.

### Defining Connectivity Reflecting Neuronal Interactions

The primary objective of this study is to extract and interpret the compressed topological principles representing interactions among neurons (see Figure 2D). Historically, many studies have sought to quantitatively characterize such interactions. Previous research in this field has termed these interaction networks as “effective networks” (Aertsen et al., 1989; Friston, 1994). Transfer entropy (TE) is an outstanding approach to estimate these effective networks. The topological architecture of networks reconstructed using this variable has been consistently explored in various studies by different research groups (Garofalo et al., 2009; Kajiwara et al., 2021; Lizier et al., 2008; Orlandi et al., 2014; Shimono & Beggs, 2015; Stetter et al., 2012; Wibral et al., 2013). Furthermore, TE has exhibited several preferred capabilities in estimation (Lungarella et al., 2007) and has known systematic relations with other information variables (Oizumi et al., 2016).

In this study, we applied a network embedding approach to effective networks previously estimated from neuronal spikes (Kajiwara et al., 2021). We utilized binarized connectivity for this purpose. For a more detailed explanation, please refer to the Supporting Information.

Before inputting into the NNE, we divided the neuron groups into smaller subsets, each containing just 100 neurons, producing 15 datasets. We then segmented these neuron groups vertically relative to the cortical surfaces (as depicted by the white lines in Figure 2C). Due to these divisions, all datasets encompass all cortical layers, and each neuron can only belong to one data group. We arranged the order of neurons based on the formula: ([cell category] −0.5) × [layer category] ([cell category] −0.5) × [layer category] to aptly express a neuron’s identity with a singular index. Here, the [cell category] is 0 for inhibitory neurons and 1 for excitatory neurons. The [layer category] provides one of four indices (1, 2, 3, 4) representing cortical layers 1–3, 4, 5, and 6, respectively.

We partitioned the 15 datasets into five subdatasets. One out of these five subdatasets served as the test data, and the remaining four subdatasets were used as training data in a fivefold validation procedure (see Figure 2E). Neurons (or nodes) with no links, either input or output, were omitted in subsequent procedures since we can infer that such neurons are effectively isolated in the slice.

### Network Embedding: Searching Minimum Expressions

#### NNE: Nonlinear automated feature extraction.

**Figure 3.**
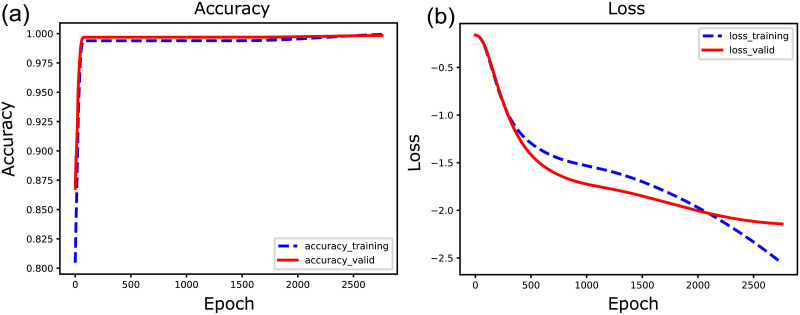
A schematic example of learning curves for training data and for validation data. The two panels (A and B) in this figure illustrate how the values of accuracy and loss change as the number of epochs increases and the NNE model undergoes training. The dotted line represents the results from the training data, while the solid line displays the results when applying the trained model to the remaining test data.

In our NNE analysis, we employed an artificial neural network with a symmetric layer architecture, comprising multiple encoders and decoder layers. Specifically, the number of units in each layer—or at each depth of the artificial neural networks—decreases linearly from the number of experimentally recorded neurons provided in the input layer. Subsequently, the number increases linearly from the middle layer to the output layer. This structure allows the design of the NNE to be governed by just two parameters: the depth of layers and the number of nodes in the middle layer (refer to Figure 3A). Initially, each layer is fully connected with the next layer but not within each layer. All layers, except for the output layer, use the rectified linear unit (ReLU) as their activation function (Brownlee, 2019). The output layer employs the sigmoid activation function to convert output values, and these values are thresholded at 0.5 to produce binary values.

The artificial neural networks were optimized using the “Adam” optimizer (Kingma & Ba, 2015). We adopted the default values for the optimizer’s parameters (such as learning rate, beta 1, beta 2, and epsilon; for further details, see the manual document for the “Adam” function in Keras at https://keras.io/api/optimizers/adam/). During the optimization phase, we minimized discrepancies between the values in the input layer and those in the output layer, quantified using a binary cross-entropy loss, in line with the standard NNE optimization scheme.

We trained the NNE model using five different combinations of datasets and evaluated the loss function’s average value. If the loss function did not decrease for 50 consecutive epochs in the training data, we deemed those 50 epochs sufficient for convergence and used the last epoch count for the final evaluation on the test data. All NNE models were implemented using the Keras deep learning platform (Chollet & Chollet, 2021; Keras. https://github.com/fchollet/keras), with a Tensorflow (www.tensorflow.org) backend.

#### Evaluation of reconstruction errors.

Reconstruction errors assess losses (or the inverse of performances) by measuring discrepancies between inputs and outputs after processing through a compressed latent space using embedding methods, including NNE. Specifically, lower reconstruction errors for embedding methods signify superior embedding performances. The NNE model’s training process is directed toward minimizing these errors. As previously mentioned, the reconstruction error in our study was quantified using the binary cross-entropy loss to optimize the NNE models. The binary cross-entropy loss is recognized as a measure more compatible with stochastic gradient descent than the mean squared error (Goodfellow, Bengio, & Courville, 2016) and is defined by the following equation:

$$\text{Loss}=-\frac{1}{N}\sum_{i=1}^{N}(y_{i}\log p_{i}-(1-y_{i})\log(1-p_{i}))$$

In this equation, *NN* denotes the number of samples in the dataset, and *y*_*i*_ represents the true label of the *i*-th sample, taking a value of either 0 or 1. *p*(*y*_*i*_) indicates the predicted probability of the *i*-th sample. The entire equation gauges the level of “discrepancy” between the true labels and the predicted probabilities. For each sample, specific terms are calculated based on whether the true label is 1 or 0. Summing these values and averaging them yield the overall loss. This loss function diminishes as the prediction aligns with the true label and augments as it deviates from it.

We also employed the binary cross-entropy loss to quantify the difference between the reconstructed signal from swapped data processed through trained NNE models and the one derived from raw data.

### Network Metrics: Interpretations of Extracted Features

#### Centrality metrics and several other network metrics.

**Figure 4.**
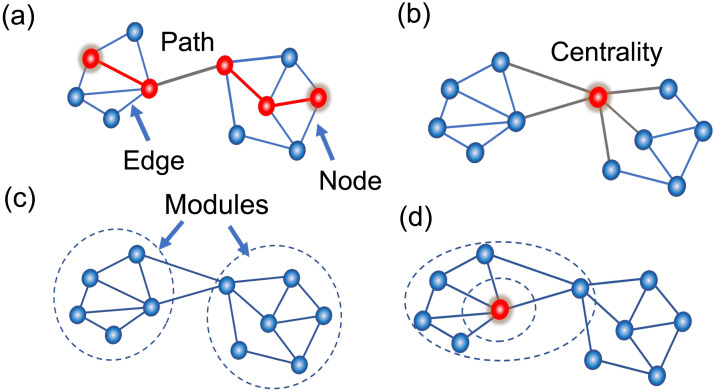
Concept figure to explain utilized network metrics: This panel (A) illustrates the concepts of nodes, edges, and paths. A node is a point connected by an edge (line), and a path is a series of paths to go from one node to another node connected indirectly. Panel B illustrates centrality. It evaluates how central a node is in the whole network from various perspectives. For example, degree centrality is evaluated based on how many nodes are connected to it, while betweenness centrality is evaluated based on how many of the shortest paths between nodes pass through it. (C) A module is a large group of nodes in the overall network. The density of connections within each module is high, while the density of connections between different modules is relatively low. (D) The ability given in common to the newly defined network metrics is to find situations where hubs are located at some path length away from the particular node of interest (red dot), or where an increase in degree can be observed.

To interpret the features automatically extracted by NNE analyses, we calculated 15 network metrics, as their quantitative meanings are clear to researchers. Since a value needs to be assigned to each cell, we focused primarily on a range of local network metrics. Among the metrics calculated, the first group includes centrality-type metrics such as degree, subgraph centrality, betweenness, closeness, and page rank. These centrality-type metrics were selected because previous studies have identified centrality or closely related measures as key features characterizing the network topology of local effective neuronal connectivity networks (Nigam et al., 2016; Shimono & Beggs, 2015; Figure 4).

The most basic centrality metric, the degree of node *i*, represents the number of nodes linked to node *i*. Given that our connection matrix is asymmetric, we used out- and in-degree in this study. Betweenness centrality considers paths connecting two different nodes: The betweenness for a node is the number of shortest paths connecting all node pairs that pass through node *i*. Subgraph centrality quantifies centrality in terms of node subsets, or subgraphs. Specifically, the subgraph centrality of a node *i* is determined by how frequently and extensively the node participates in different subgraphs within a network (Estrada & Rodríguez-Velázquez, 2005). Here, subsets are defined as closed walks passing through the focal node. Core number refers to the largest value *k* of the *k* cores that include that node (Batagelj & Zaversnik, 2003). Page rank is a centrality metric based on the number of input links to a node (Brin & Page, 1998). Closeness centrality of a node is proportional to the inverse of the sum of shortest path lengths (Bavelas, 1950).

To complement these centrality metrics, we also calculated the participation coefficient, a metric defined based on community structure. The community structure was determined using the Louvain algorithm, with an optimization process that incorporated fine-tuning across iterations to achieve a partially optimized community structure. Although the participation coefficient is somewhat similar to subgraph centrality, it quantifies how frequently a node engages in various communities. These values depict the overarching network architectures since the basic node groups are large communities, not small subgraphs. Furthermore, the cluster coefficient of a node *i* (denoted as ***C***_***i***_) is given by:

$$C_{i}=2e_{i}/k_{i}\left(k_{i}-1\right)$$

Here, ***k***_***i***_ is the degree of node *i* and *e*_*i*_ is the number of links connecting the neighboring nodes of node *i* (Watts & Strogatz, 1998). The local efficiency of a node is simply the mean of the inverses of the shortest path length with all other nodes.

We used the “NetworkX” (Hagberg et al., 2008), “python-louvain,” and “Brain Connectivity Toolbox” (Rubinov & Sporns, 2010) Python modules to calculate these representative network variables (Type1 and Type2 Table 1).

**Table 1.** Subcategories of network metrics: Network metrics are categorized into three types

Type 1	Centrality	Degree (out-degree),
		InDegree (in-degree),
		Betweenness,
		Subgraph centrality
		Core number
		Page rank
		Closeness
Type 2	Noncentrality	Local efficiency
		Participation coefficient
		Cluster coefficient
Type 3	New metrics	Indirect-adjacent degree
		1st neighbor hub ratio
		2nd neighbor hub ratio
		3rd neighbor hub ratio
		4th neighbor hub ratio

*Note*. The first type is the commonly used metric of centrality, which has been widely examined because past studies have shown that nodes with exceedingly high centrality exist in local circuits of the nervous system. The second type is a commonly used metric that is somewhat different from centrality. Although local efficiency is similar to betweenness centrality, it evaluates the ability of individual nodes to shorten paths in a more local network structure (cluster). The participation coefficient evaluates whether a node is able to generate information flow across modules in the whole network (Figure 4C). The third type is a new set of network metrics, which were originally defined in this study to compensate for the characteristics that the centrality metrics might overlook. Refer to the main manuscript for their explanation.

#### Designing new network metrics.

We were unable to adequately interpret several compressed features of NNE using the centrality metrics and other network metrics. As a result, we designed new network metrics inspired by the NNE compressed features that traditional network metrics could not fully explain. Specifically, we introduced two new network metrics:

The first new metric is termed the “*N*-th neighbor hub ratio.” This metric quantifies the ratio of hub nodes among nodes that are *N*-steps apart from node *ii*. Here, hub nodes are defined as nodes possessing the top 20% highest degree (Barabási, 2016; Yu et al., 2007). We evaluated cases of *n* = 1, 2, 3, and 4 for all nodes in the neural connectivity maps, considering the matrix size. These metrics draw inspiration from several past studies addressing the “second neighborhood problem” (Brantner, Brockman, Kay, & Snively, 2009).

Additionally, to capture and characterize more effectively, we introduced another type of metric. The second new network metric is labeled the “relative indirect-adjacent degree.” This metric employs the equation <D2(i)>/<D1(i)>, where <D1(i)> is mean value of degrees among neighbor nodes of a given node *i* and <D2(i)> is the mean value of degrees among nodes two steps away from node *i*. As a result, this metric assigns a high score to a node that is two steps away from hub nodes.

The indirect-adjacent degree can be considered a neighborhood generalization of the disassortativity parameter, which describes a node’s degree in relation to its neighbors’ degrees (Kartun-Giles & Bianconi, 2019).

It is noteworthy that existing network metrics primarily encompass many centrality metrics of type 1. The two newly introduced metrics are specifically crafted to capture characteristics that the centrality metrics overlook. These variables, in fact, emphasize nodes somewhat distant from the hub. Furthermore, in systems where hubs are typically situated at the centers of modules, these metrics are anticipated to underscore areas near the boundary more than those near the central module.

One of these metrics successfully captured an NNE feature uncorrelated with centralities. For a more direct observation of their distributions, refer to Figure 6E and 6F and Figure 7E–G.

### Mutual Information

We employed mutual information to assess the relationships between NNE’s compressed features and 10 representative network metrics, as well as five new network metrics (Studholme et al., 1998). In order to calculate the *p* value for resultant mutual information values, we performed permutation tests with 100 iterations. Furthermore, for multiple testing correction, we calculated the false discovery ratio (FDR) for resultant *p* values by the Benjamini-Hochberg method. In this study, to emphasize variations among the NNE features, we normalized the mutual information values for each network metric using min-max scaling. Without this normalization of mutual information values across the 13 NNE features for each network metric, we would primarily observe the commonality among the NNE features. For instance, the 13 NNE features exhibited strong relationships with a few network metrics (e.g., indirect-adjacent degree, subgraph centrality, local efficiency, etc.). We calculated mutual information using modified codes from https://github.com/mutualinfo/mutual_info. For multiple testing correction, we used R language Version 4.2.0 and the p.adjust function.

### Networks by the Barabási-Albert (BA) Model

We generated 14 networks composed of 1,000 nodes based on the BA model. The connectivity density was 40.0% ± 6.2%. We used NetworkX Python packages to generate the BA networks.

## RESULTS

### Compressing With NNE

First, we optimized the architecture of the deep neural networks following the method outlined in Evaluation of reconstruction errors section. The input networks represent interactions among neurons (Kajiwara et al., 2021).

**Figure 5.**
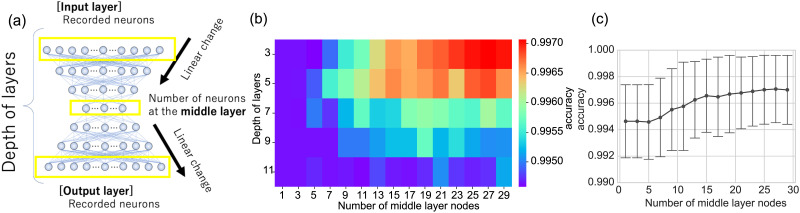
The network architecture of deep neural networks and loss in the learning process. (A) The architecture of neural networks utilized for deep neural networks in this study. The number of units (artificial neurons) gradually decreases in a linear trend from the number of units of the real neural network to a much fewer number of units located at the middle layer, and gradually increases in a linear trend again toward the output layer. The key two parameters describing this network architecture are (Aertsen et al., 1989) the depth of layers and (Bales & Johnson, 2006) the number of units at the middle layer. (B) The loss function, binary cross entropy, is mapped on the two-dimensional map of “depth of the layers” (“*Depth*”) and “number of middle layer units” (“*Middle Size*”). The binary entropy was averaged for seven data samples. (C) Accuracy only on the section at depth layers equal 3 is shown. We especially focus on the parameter region where the “number of middle layer units” is 13 shown as the inserted allow.

The number of units, or artificial neurons, at each layer decreases linearly from the input layer, which contains the number of experimentally recorded neurons, to the middle layer, which contains the minimum number of neurons (Figure 5A). After this compressing phase, the number of units increases linearly from the middle layer to the output layer, which has the same number of units as the input layer. Due to this design, the architecture of the deep neural networks can be characterized solely by two parameters: *the depth of layers* (*Depth*) and *the number of neurons in the middle layer* (*Middle Size*; Figure 5A).

Using this basic architecture, we observed loss functions for depth values ranging from 3 to 11 and middle layer sizes between 1 and 29 (Figure 5B). We conducted a fivefold cross-validation, allocating 12/15 of the data for training and 3/15 for testing. For detailed learning and testing processes, please refer to the Materials and Methods section.

After completing sufficient learning steps, which were adaptively chosen based on the stabilization of the loss function, we assessed the cross entropy for the training data. The colormap of the loss function for the test data (Figure 5B) suggests that loss gradually increases as the network architecture deepens. This could be due to the gradient vanishing, even though we employed the ReLU function, known to mitigate this issue.

The initial wide-range parameter survey indicates that NNE can compress complex network sizes to 13% of their original node or unit count while maintaining a stable accuracy for all datasets. Based on this survey, we chose the parameter pair (Depth, Middle Size) = (3, 13) for the following reasons: The Depth value of 3 appears optimal because the accuracy is higher than for deeper networks (>4). The second parameter was selected since the accuracy values plateaued around a Middle Size of 13 when the Depth was set to 3 (indicated with an arrow in Figure 5C). As shown in Table 2, for Depth of 3, it is pertinent to note here that the value of accuracy for the Middle Size of 13 is significantly higher than that of 1 (*p* < 0.1, Weltch’s *t* test with multiple testing correction of Benjamini-Hochberg method), while there were no significant difference in accuracy (loss) between NNE with the Middle Size of 1 and that with 3 to 11, and firstly became significant with the size of 13. It is known that the local circuits of neurons exhibit an architecture that is nearly scale free. Therefore, we verified whether the parameter selection made in this study could also be applied to the general BA model. As a result, similar parameters were naturally selected (Supporting Information S1).

**Table 2.** Difference in accuracy between # of middle nodes = 1 and # of middle node = *m*

*m*	Original (non_transposed)	Original (transposed)	AB model
3	n.s.	n.s.	n.s.
5	n.s.	n.s.	n.s.
7	n.s.	n.s.	n.s.
9	n.s.	n.s.	n.s.
11	n.s.	n.s.	n.s.
13	*	**	*
15	**	***	n.s.
17	*	**	n.s.
19	**	**	*
21	***	**	*
23	**	***	*
25	**	**	*
27	**	***	*
29	**	***	*

*Note*. *, **, ***, and n.s. indicate difference in accuracy between # of middle nodes = 1 and # of middle nodes = *m*, that is, *, **, and *** indicate *p* values with Bonferroni’s multiple testing correction < 0.1, < 0.05, and < 0.01, respectively. n.s. is nonsignificant.

We refit the NNE with Middle Size = 13 and Depth = 3 by using all the networks. Given that the input matrix size is 100 × 100, we ultimately obtained 13 vectors from the refitted NNE model, each with 100 components. These are the features we have been discussing.

In order to benchmark the NNE with a simpler machine learning algorithm, we compared accuracy and error rate (1 − accuracy) of the refitted NNE with a principal component analysis (PCA) fitted with all the networks. The accuracy and error rate of the NNE are better than those of PCA (see Supporting Information Table S1).

### Interpreting Compressed Features With Centrality Measures, and Other Common Network Variables

**Figure 6.**
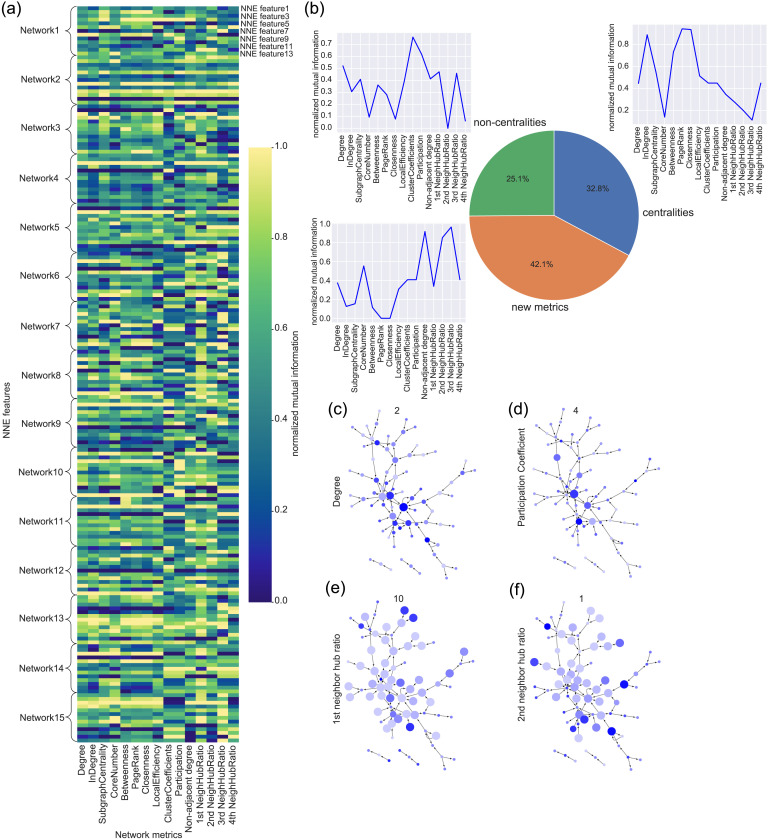
Comparing NNE’s features in comparisons with network variables: (A) This colormap shows normalized (over 15 networks) mutual information between 13 NNE’s features and 15 network variables. (B) The pie chart represents the ratio of centrality type, noncentrality type, and newly designed network metrics better explaining NNE features than the other metrics. The inserted three line graphs represent three examples of normalized mutual information between an NNE feature and the 15 network metrics, that is, the line graph associated with the “new metric” category of the pie chart indicates that the new metric played a fairly good role to interpret the automatically extracted features. (C–F) Example of network visualizations to show relationship between NNE features and representative network metrics. Marker sizes reflect network metrics, and marker colors show NNE’s features written in the individual panels. For example, C is an example of network visualization with expressing degree centrality as marker sizes and second NNE’s feature as the marker colors. D is an example between participation coefficient and fourth NNE’s feature. Similarly, E is an example between the first neighbor hub ratio and the 10th NNE’s feature, and F is one between the second neighbor hub ratio and the first NNE’s feature.

In this study, we calculated five centrality network variables: degree, subgraph centrality, betweenness centrality, core number, and page rank. To focus on variations among the NNE features from the refitted NNE model with Middle Nodes = 13 and Depth of layer = 3, we examined the normalized mutual information between the NNE features and these network metrics (Figure 6A and Supporting Information S2A; refer to the Materials and Methods section for details). We specifically sought network metrics that exhibited a higher correlation with any of the NNE features, based on normalized mutual information values.

Among the NNE features, 25.1% were better explained by centrality variables compared with other network metrics (Figure 6B). For a more intuitive representation of features that align with the centrality metric, we visualized a network in which the sizes of the markers reflected the second feature from the NNE model (Figure 6C). As evident from the results, larger markers naturally occupy the center of the network.

Besides centrality variables, we also considered other common noncentrality network variables, such as local efficiency and participation coefficient. These metrics provided a better explanation for 32.8% of NNE features compared with other network metrics (Figures 6B and 6D).

### Explanation Ability by Adding Originally Designed Metrics

Can we uniquely characterize the compressed features obtained from the NNE model using metrics beyond the commonly used network variables? To address this, we introduced two new types of network metrics (Figures 6A and 6B and Supporting Information Figure S1A). The first metric, named “indirect-adjacent degree,” calculates the ratio between the degrees of neighboring nodes of a given node *i* and the degrees of nodes two steps away from node *i*. The second metric, termed “*N*-th neighbor hub ratio,” quantifies the proportion of hub nodes—those with degrees in the top 20%—among nodes that are *NN*-steps away from a given node *i* (Barabási, 2016; Yu et al., 2007).

While we anticipated that these metrics might capture characteristics not significantly tied to common centrality- and noncentrality-type network metrics, they notably succeeded in characterizing a high percentage of features. Specifically, the new network metrics more effectively explained the remaining 42.1% of the NNE features than the traditionally used network metrics (see Figure 6B).

Let us visualize two networks where the 10th and first NNE features dictate the marker sizes (Figures 6E and 6F). These illustrations show that the largest marker sizes tend to be one or two nodes away from hubs. This characteristic aligns with our new metrics, namely, the neighbor hub ratios. In other visualizations, the centrality metric tends to enlarge markers near the network’s center, whereas the new metric enlarges markers further from such points.

### Transposed Case

Next, we utilized the transposed matrices, in which the rows and columns of the matrices were swapped, as input to the NNE. Contrary to our previous analysis using nontransposed data, this transposed case focuses on analyzing the output signals for a specific neuron. From the perspective of real neurons, this distinction boils down to whether we prioritize input connections from dendrites or outputs from axons; both interpretations carry distinct physiological implications.

**Figure 7.**
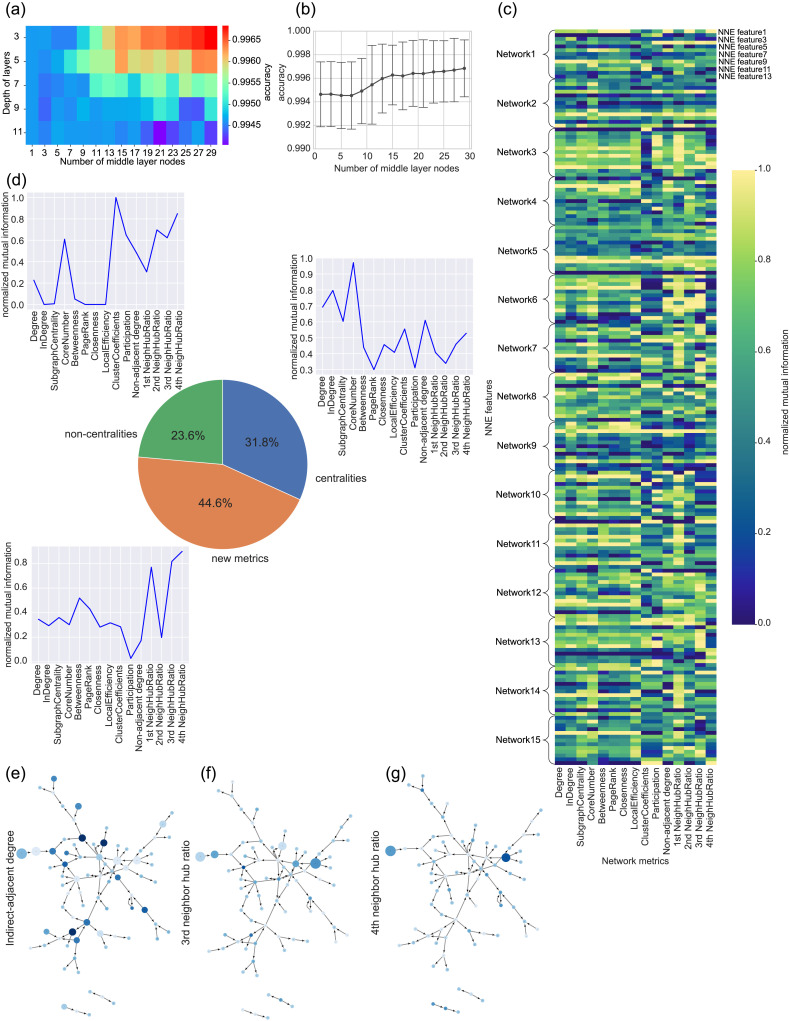
Evaluations of transposed matrices: (A) The influences of two parameters, the number of middle layer nodes and the depth of layers, of NNE’s architecture. This panel is the same map with Figure 5B except that we utilized the transpose matrix as the input to the NNE. B is also basically the same as Figure 5C except the input adjacency matrices were transposed. C is the colormap of mutual information between NNE’s features and network metrics including originally designed network variables here. (D) The pie chart represents the ratio of the centrality type, noncentrality type, and newly designed network metrics better explaining NNE features than the other metrics. The three line graphs represent normalized mutual information for the three representative NNE features. E shows three examples of network visualizations, in which marker sizes express network metrics and marker colors express NNE’s features written in the individual panels at the positions of the *y*-axis and the title.

Interestingly, our results from this transposed analysis mirrored the patterns observed in the nontransposed scenario (Figure 7 and Table 2). For transposed networks, the accuracy and error rate of the NNE are also better than those of PCA (see Supporting Information Table S1). A notable commonality is that the new network metrics more accurately explained 44.6% of the NNE features compared with the traditional network metrics (Figure 7D). Especially in this context, the indirect-adjacent degree and the third and fourth neighborhood hub ratios correlated with several NNE features, suggesting its potential in capturing essential characteristics unearthed by NNE (Figure 7E–G).

In conclusion, not only do we observe similarities in relationships with network metrics akin to the nontransposed case, but there also seems to be a trend where the loss is further reduced when using transposed data compared with nontransposed data.

### Evaluation in Swapped Networks

From our analyses thus far, the extent of the NNE model’s generalization capability and its proficiency in extracting specialized information from real data remains ambiguous. To assess this quantitatively, we evaluated the model’s performance when presented with swapped data. Specifically, we were curious if errors would increase when the NNE model, trained on real data, was subjected to data with altered connections. Given the nature of actual neural connections, it is plausible to consider two situations, situation (a) where the total number of connections remains constant, but the specific interneuronal links are reshuffled, and situation (b) where the links are reshuffled randomly without preserving the total number of connections of each node. These help determine if the NNE is sensitive to the intricate structure of actual neuron-to-neuron connections, which are vital for specific information-processing tasks.

**Figure 8.**
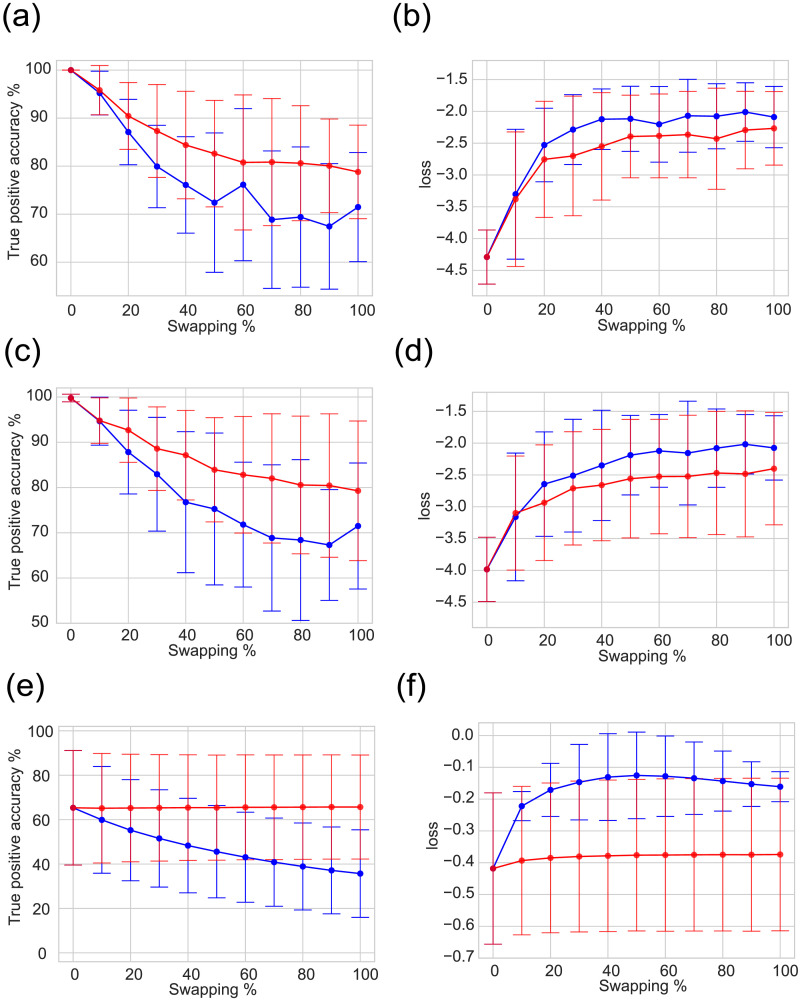
The evaluation of generalization ability to swapped data. When the degree of swapping to the network was gradually increased as a percentage of the number of connections that originally existed, we evaluated how much error the restored network had by using decreases in TP accuracy (TP accuracy = 100 * TP/(TP + false negative)) as well as in the loss value. Error bars are standard deviation of networks. (B) TP accuracy for original neural networks, (C) loss for original network, (D) TP accuracy for transposed networks, (E) loss for transposed networks, (F) TP accuracy for networks by BA model, and (G) loss for networks by the BA model. Red and blue lines represent results for swapping edges with and without degree preservation, respectively.

For situation (a), the swapped data were crafted by randomly relocating the connected sections of the original connection matrix to different positions within the matrix, while ensuring that the out-degree histogram (both density and sequence) remained consistent (Váša & Mišić, 2022). We incrementally increased the proportion of reshuffled connections, exploring ratios of 0%, 10%, 20%, 30%, 40%, 50%, 60%, 70%, 80%, 90%, and 100% (Figure 8).

The findings revealed that as connections were swapped, information loss ensued, evidenced by a gradual decrease in reconstruction accuracy, for both of edges swapping with/without degree preservation. In the networks by the BA model, true positive (TP) accuracy and loss decreases more rapidly for swapping without degree preservation than for that with degree preservation. The results indicates that the NNE models are able to capture structures other than scale-free architecture.

## DISCUSSION

In data interpretation, reducing researcher bias is paramount as conclusions can be heavily influenced by the metrics chosen. As technological advancements lead to an exponential increase in the number of simultaneously measurable neurons, the development of automatic compression technology will become increasingly crucial (Hong & Lieber, 2019; Stevenson & Kording, 2011).

Building on this need, our study introduces a “NNE” methodology. This approach leverages the advancements in artificial neural networks to automatically extract features from network architectures, minimizing subjective researcher bias (LeCun et al., 2015; Samek et al., 2021; van der Laak et al., 2021).

In our study, we employed a data-driven approach using NNE to extract features, which we then compared with various network metrics to interpret extracted features. The network metrics considered were classified into centrality metrics, existing noncentrality metrics, and newly developed metrics such as indirect-adjacent degree and neighbor hub ratios. These new metrics specifically aim to identify scenarios where neurons’ several steps apart from a particular neuron correlate with hubs or exhibit a higher degree than their adjacent neighbors.

In this comparison, our new network metrics such as indirect adjacency and adjacency-to-hub ratios showed better explanation ability about 40%–45% than centrality metrics and existing noncentrality metrics. This provides insight that there are inherent features that cannot be explained solely by simple representative characteristics like hubs or clusters. Similar results were observed in transposed networks, confirming the robustness of our findings.

We also demonstrated that a similar compression ratio can be achieved with a BA model consisting of 1,000 nodes, similar to the network model given from our experimental data. Although the compression ratio in our neural data was significantly higher than that achieved with PCA, the compression ratio in the BA model was comparable to that obtained with PCA. This suggests that our NNE methodology has superiority in performing compression of nonrandomness beyond just degree sequence information.

Furthermore, it was observed that NNE reconstruction performance significantly deteriorates with swapping that does not preserve the degree sequence, compared with swapping that does. Additionally, in the BA model, no degradation in reconstruction performance was observed with swapping that does not preserve the degree sequence. These results once again demonstrate that in our experimental data, nonrandomness beyond degree sequence information can be found.

In conclusion, the compression technique employed in this study not only demonstrated high compressive capability but also provided an unbiased method that highlights the presence of features beyond readily understandable perspectives such as hubs and scale free in the interpretation of compressed features.

These results revealed the technique’s potential ability to illuminate complex characteristics beyond the network architecture whose interpretation is relatively simple or well designed.

The number of neurons that can be recorded will definitely increase in the future, and the importance of approaches like NNE will grow as well. Network embedding methods are already being applied to brain-wide connectomes (Rosenthal et al., 2018), and as we consider networks with neurons as elements, the required elements will multiply several-fold. It is also important to compare the compressibility of connectivity between brain regions, as demonstrated in this study (Matsuda et al., 2022; Nakajima et al., 2023).

If we survey wide analysis methodologies, there are various network embedding methods, especially those rooted in deep learning. Deep autoencoder-based methods like Structural deep network embedding, stacked denoising autoencoders, and signed network embedding have emerged as potent tools for feature extraction from complex networks (Vincent et al., 2010; Xu et al., 2021). These methods have been employed for community detection (Ye et al., 2018), node clustering (Yang et al., 2019), and even drug-target gene inference for diseases like Alzheimer’s disease (Tsuji et al., 2021). These methods are inevitably necessary longer analysis times compared with PCA, but by accelerating through improvements in associated deep neural network analysis algorithms, such as batch processing and quantization, these methos will practically become more adaptable for larger datasets.

The future beckons further exploration of these methods will finally offer deeper insights into brain states and various disease conditions. The key Python codes are shared on GitHub at https://github.com/ShimonoMLab/NNECode.

## ACKNOWLEDGMENTS

M.S. is supported by several MEXT fundings (19H05215, 20H04257, 21H01352, 23K18493) and the Leading Initiative for Excellent Young Researchers (LEADER) program, as well as grants from the Uehara Memorial Foundation. The MRI experiments of this work were performed in the Division for Small Animal MRI, Medical Research Support Center, Graduate School of Medicine, KU, Japan. We warmly acknowledge Takuma Toba, Tatsuya Tanaka, Hirohiko Imai, and all the support of the Hakubi Center to establish this study. The supercomputing resource was provided by Human Genome Center, the Institute of Medical Science, The University of Tokyo (https://sc.hgc.jp/shirokane.html).

## SUPPORTING INFORMATION

Supporting information for this article is available at https://doi.org/10.1162/netn_a_00424.

## AUTHOR CONTRIBUTIONS

Arata Shirakami: Data curation; Formal analysis; Methodology; Software; Validation; Writing – review & editing. Takeshi Hase: Conceptualization; Formal analysis; Methodology; Supervision; Validation; Visualization; Writing – original draft; Writing – review & editing. Yuki Yamaguchi: Data curation; Formal analysis; Software; Validation; Visualization. Masanori Shimono: Conceptualization; Data curation; Formal analysis; Funding acquisition; Investigation; Methodology; Project administration; Resources; Software; Supervision; Validation; Visualization; Writing – original draft; Writing – review & editing.

## FUNDING INFORMATION

Masanori Shimono, MEXT fundings, Award ID: 19H05215, 20H04257, 23K18493. Masanori Shimono, LEADER program, Award ID: MEXT funding. Masanori Shimono, Uehara Memorial Foundation (https://dx.doi.org/10.13039/100008732).

## Supplementary Material



## TECHNICAL TERMS

Centrality: Centrality is a measure that indicates the importance or influence of a node (vertex) within a network.
Clustering coefficient: Clustering coefficient is a measure that quantifies the degree of connectivity among neighboring nodes in a network (graph). Specifically, it indicates the extent to which the nodes adjacent to a given node are connected to each other.
Participation coefficient: Participation coefficient is a measure that quantifies the extent to which a node in a network is broadly involved with different modules, rather than being connected only within a single module.
Network embedding: Network embedding refers to techniques that transform the structural information and characteristics of complex networks (graphs) into low-dimensional vector representations that are more easily processable by machine learning algorithms.
Multi-electrode array: Multi-electrode array is a recording method used to measure the electrical activity of neurons with multiple electrodes. By using many electrodes, it is possible to record the activity of multiple cells simultaneously.
Cortex: In this article, the term “cortex” mainly refers to the neocortex, the most recently evolved part of the brain, which covers its surface in a sheet-like region.
Cortical layers: The cortical sheet consists of six layers, each referred to as a “layer,” a term used in physiology. Notably, in this paper, these layers differ from the ones stacked from input to output in artificial deep networks.
Module: Module, also called a community, is a term referring to a large group of nodes within a network (graph) that are relatively more densely connected to each other compared with their connections with nodes in other groups.
Indirect-adjacent degree: Indirect-adjacent degree is a newly developed network metric in this paper. It measures how many high-degree nodes are located two nodes away from a given node, compared with the nodes directly connected to it.
