# Supplementary material for: Neural network embedding of functional microconnectome
Source: Netw Neurosci. 2025 Mar 5;9(1):159–80. doi: 10.1162/netn_a_00424 (PMC11949542; doi:10.1162/netn_a_00424)
Supplement: Supplementary file 1 [file netn-9-1-159-s001.pdf]

Supplemental materials

Connectivity estimation and E/I categorization

In this study, we first classified neurons into inhibitory and excitatory types. Subsequently, we estimated the presence or absence of connections and calculated the strength of connections for each neuron type. It's crucial to classify neurons before determining connectivity, as the shuffled null data and the real data for excitatory and inhibitory synaptic connections need to be compared separately. We utilized Transfer Entropy (TE) to estimate connectivity and Sorted Local Transfer Entropy (SLTE), a modification of TE, to classify neurons into either inhibitory or excitatory (E/I) categories. Our method primarily builds upon the approach developed by Kajiwara et al., 2021. The source code is available on GitHub (https://github.com/Motoki878).

TE, employed for connectivity estimation, evaluates information transfer between distinct variables, circumventing false correlations and confounding factors [Okatan et al. 2005] (Supple. figure 1-(b)). The relationship between structure and function [Hlavackova, Schindler et al. 2009; Stetter et al. 2012; Honey et al. 2007; Ito et al. 2011; Timme et al. 2016], the consistency of topology with patch-clamp experiments, the identification of hubs and modules [Shimono, Beggs, 2014], and the demonstration of a log-normal distribution of connection weights [Nigam et al., 2016] all underscore the efficacy of TE. Additionally, connection weights have been observed to approximate a logarithmic distribution. Thus, TE offers advantages in terms of its theoretical formulation and its alignment with physiological data [Lizier et al. 2008; Vicente et al. 2011; Wibral et al. 2013].

Moreover, SLTE, which classifies neurons into inhibitory and excitatory types, operates similarly to TE. However, SLTE categorizes local transfer entropy based on the positive or negative signs of different interactions [Lizier et al., 2008]. Due to its ability to sort by sign, SLTE can discern between excitatory and inhibitory interactions of presynaptic spike events in relation to postsynaptic spike events, leveraging the opposite signs of local transfer entropy for excitatory and inhibitory interactions [Lizier et al. 2008; Goetz, Lai, 2019].

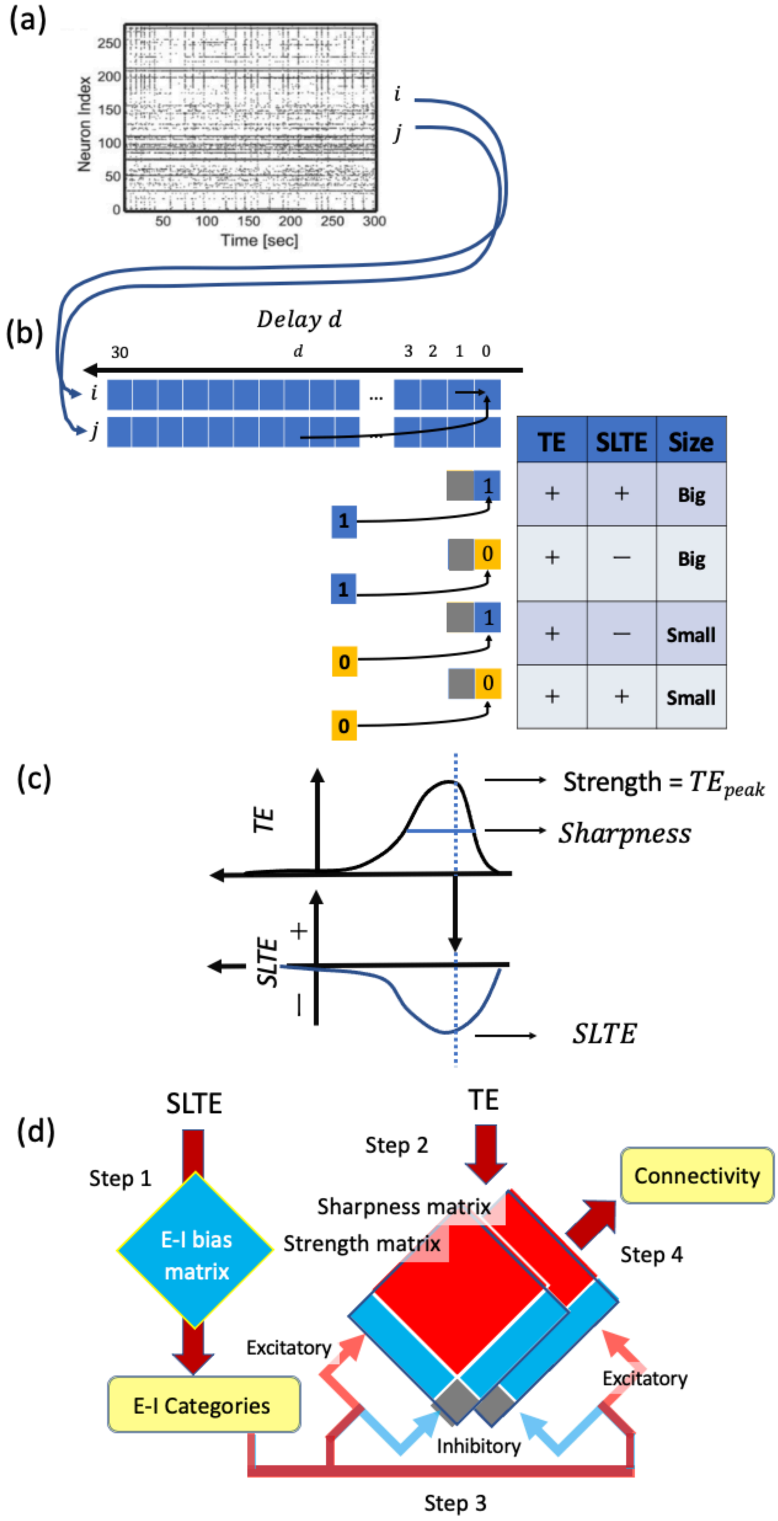

Supplemental figure 1. Estimation of E/I categories and intercellular connections from spike data:
(a) was an example of spike data displayed as a Raster plot. Neurons i and j were extracted from this large number of neurons.
(b) Then, the influence of other neuron j on the main neuron i was evaluated by transfer entropy. The effects of the combination of the activity state of neuron i at delay 0 and the activity state of neuron j at delay d on the TE and SLTE values were summarized in a table with positive and negative values. The magnitude of

the effect was expressed as "big" or "small".
(c) TE was obtained as a function TE(d) that depends on delay, and from TE(d), the peak value Strength and the peak Sharpness were calculated. The value of SLTE at the delay where TE takes the peak was also extracted.
(d) is a flowchart describing the overall flow of cell categorization and connectivity estimation after the SLTE and TE are prepared. Step 1: The E-I bias matrix was obtained from the peak SLTE values, from which the E-I category for each neuron was determined; Step 2: Strength and Sharpness were obtained as a matrix from the TE values for all combinations of neurons; Step 3: The matrix was classified into four connection types based on the E-I categories. Step4: Finally, the presence or absence of connections and the strength of the connections were obtained by comparing them to the shuffled data for each of those categories.

More information about connectivity estimation

The transfer entropy (TE) employed for connection estimation gauges the improvement in prediction accuracy for the activity of neuron I when information on the spiking activity of neuron J is incorporated. This is in contrast to prediction accuracy that relies solely on neuron I's past activity (Fig. 6-a). The equation for TE in this study is given by:

$$TE_{J\to I}(d) = \sum_{i_t, i_{t-1}, j_{t-d}} p(i_t\,, i_{t-1}, j_{\ t-d}) \log\left(\frac{p(i_t|i_{t-1}, j_{t-d})}{p(i_t|i_{t-1})}\right) \qquad (1)$$

This equation computes the expected value of the local transfer entropy $\log\left(\frac{p(i_t|i_{t-1}, j_{t-d})}{p(i_t|i_{t-1})}\right)$ across all i_{t-1}, j_{t-d} and i_t [Schreiber, 2000]. The variables i or j are indexed by time to denote if neuron *I* or *J* is active at a given time bin. They are assigned a value of 1 when spiking and 0 otherwise.

The time bin size was fixed at 1 ms, typical for spiking data. We assessed the improvement in prediction accuracy when neuron *J's* time bin at $t - d$ ms was used in conjunction with neuron *I's* time bin at $t - 1$ ms to predict the state of neuron I's latest time bin at t ms (Suppl. figure 1-b). Multiple past time bins for neuron *J* don't notably alter the prediction accuracy as represented by TE(d) [Shimono and Beggs, 2014; Nigam et al., 2016] (Suppl. figure 1-c).

A pronounced and sharp peak in TE(d) arises when there's a direct interaction stemming from synaptic connections from neuron *J* to neuron *I* [Berry, Pentreath, 1976]. Here, "Strength" denotes the peak value of TE(d), and "Sharpness" is defined by the subsequent equation (Suppl. figure 1-c) [Kajiwara et al., 2021; Nigam et al., 2016; Shimono, Beggs, 2014]:

$$Sharpness = \frac{\sum_{d=0}^{d=d_{peak}+\tau} TE(d)}{\sum_{d=0}^{d=30} TE(d)} \qquad (2)$$

In equation (2), the denominator's range for Sharpness is set at 30 ms, which is sufficient for electrical signals to transmit within the brain region under observation [Mason et al, 1991; Swadlow, 1994]. The numerator ranges from the standard deviation of all neuron pairs to τ = 4 ms.

Shuffled spike arrays were derived from the raw data by swapping presynaptic neuron spikes into spike-free bins within a ±10 ms range (Suppl. figure 1-d) [Hatsopoulos et al., 2003]. The TE from this is denoted as $TE_{j\to i}^{shuffle}$, while the TE from the original data is termed $TE_{j\to i}^{real}$. By juxtaposing the raw connectivity matrix with the matrix generated from 100 shuffled spike series, we identified connections with pronounced and sharp peaks in TE(d)TE(d) within the Strength-Sharpness space. The connection strength was defined as $IT_{j\to i} = TE_{j\to i}^{real} - TE_{j\to i}^{shuffle}$ for the shuffled data. This connection strength has been consistently assessed against EPSPs measured through patch clamp recordings [Shimono, Beggs, 2014]. For further details, please refer to previous studies [Kajiwara et al, 2021; Nigam et al, 2016; Shimono, Beggs, 2014].

More information about cell categorization

The Sorted Local Transfer Entropy (SLTE) used to classify neurons as inhibitory or excitatory (E/I) is mathematically defined as:

$$SLTE_{J\to I}(d) = \sum_{i_t, i_{t-1}, j_{t-d}} p(i_t, i_{t-1}, j_{t-d})(-1)^{(i_t - j_{t-d})} \log\left(\frac{p(i_t|i_{t-1}, j_{t-d})}{p(i_t|i_{t-1})}\right) \quad (3)$$

In SLTE, local transfer entropy is modulated by the factor $(-1)^{(i_t - j_{t-d})}$. Given that each neuron's events, i and j, can be either spiking (1) or inactive (0), this factor equates to +1 when both i_t and j_{t-d} are synchronous, and -1 when they differ (Fig. 6-b). Consequently, for excitatory interactions, the local transfer entropy is additive, whereas for inhibitory interactions, it is subtracted. This sorting mechanism ensures that the aggregate value is positive for excitatory interactions and negative for inhibitory ones. It's noteworthy that, in this approach, the information amount provided by $p(i_{t-1} = 0, j_{t-d} = 1)$ for a rare event $i_t = 1$is substantially greater than that given by $p(i_{t-1} = 1, j_{t-d} = 1)$ for a frequent event $i_t = 0$ (Suppl. figure 1-b).

To emphasize the time frame of direct synaptic influence, we selected SLTE values corresponding to a delay d at which TE shows a peak (Fig. 6-c). Henceforth, this peak SLTE value will be succinctly termed the *E-I bias*.

After discarding the lower 10% to retain a high signal-to-noise ratio, the E-I bias was

ranked based on the peak TE values, $TE_{nor} = TE_{peak}/(FR \cdot log\,(FR)\ + (1 - FR) \cdot log(1 - FR))$, normalized to counteract the firing rate effect. Within the two-dimensional space of this sum and the logarithm of the per-neuron firing rate, we discerned relevant clusters using hierarchical clustering under the Ward variance minimization algorithm. Neurons in clusters commonly located on the positive side of the SLTE sum were labeled excitatory, in line with Dale's principle [Dale, 1934].

In Kajiwara et al. 2021, we verified this method's high classification performance for both computational models and in vivo data. We found that comparable classification performance can be achieved with SLTE alone. However, adding the firing rate as the second axis slightly improved the classification over using just SLTE.

To summarize, we outline the combination of TE-based connection estimation and SLTE-based neuron classification in the following four-step procedure (Suppl. figure 1-d):

1. From SLTE, classify neurons into excitatory and inhibitory categories using the E-I bias matrix.
2. Using TE, compute both the strength matrix (a matrix of peak intensities related to delay) and the sharpness matrix (a matrix representing peak sharpness relative to delay).
3. Decompose these matrices into four categories: excitatory-excitatory, excitatory-inhibitory, inhibitory-excitatory, and inhibitory-inhibitory.
4. Determine connectivity and connection strength by comparing with shuffled null data for each of the four connection types.
